# Factors affecting cervical cancer screening among Yemeni immigrant women in Klang Valley, Malaysia: A cross sectional study

**DOI:** 10.1371/journal.pone.0290152

**Published:** 2023-12-15

**Authors:** Sarah Al-Oseely, Rosliza Abdul Manaf, Suriani Ismail

**Affiliations:** Department of Community Health, Faculty of Medicine & Health Sciences, Universiti Putra Malaysia, Serdang, Malaysia; University of Jos Faculty of Medical Sciences, NIGERIA

## Abstract

**Introduction:**

Cervical cancer is a significant public health problem for women worldwide. It is the fourth most frequent cancer in women globally. While early detection of cancerous lesions through screening tests leads to a better prognosis and a better chance of being cured, the number of people who go for screening is still low, especially for groups that are marginalized, like immigrant women.

**Objective:**

The purpose of this study was to identify cervical cancer screening practices and factors influencing screening status among Yemeni immigrant women living in the Klang Valley, Malaysia.

**Method:**

A cross-sectional study among 355 randomly selected respondents between the ages of 20 and 65 was conducted through an online survey. A questionnaire was sent directly to the participants via WhatsApp. The analysis was conducted using SPSS 25 with a significance level of 0.05. It included descriptive analysis, chi-square and multiple logistic regression.

**Results:**

The response rate was 59%, with the majority of the respondents being married and between the ages of 35 and 49. Screening was reported at 23.1% in the previous three years. The final model revealed that age group 50–65 years (AOR = 5.39, 95% CI: 1.53–18.93), insurance status (AOR 2.22, 95% CI = 1.15–4.3), knowledge (AOR = 6.67, 95% CI = 3.45–12.9), access to health care facilities (AOR = 4.64, 95% CI = 1.29–16.65), and perceived barriers (AOR = 2.5, 95% CI = 1.3–4.83) were significant predictors of cervical screening uptake among Yemeni immigrant women in Malaysia (p<0.05).

**Conclusion:**

According to the results, cervical cancer screening was found to be low among Yemeni immigrant women. The predictors were age group 50–65 years, insurance status, knowledge, access to health care facilities and perceived barriers. Efforts to enhance immigrant women’s participation in cervical cancer screening must tackle barriers to access to healthcare services as well as expand cervical cancer screening education programs.

## Introduction

According to the World Health Organization, cervical cancer is the fourth most common type of cancer in women [1].

Cervical intraepithelial neoplasia and cervical cancer are mainly caused by sexually transmitted Human Papilloma Virus (HPV) infection. The people who are most at risk of contracting the virus are those who have had several sexual partners, those who have been exposed to the virus, or whose partners have had several sexual partners [2].

Cervical cancer is a potentially avoidable condition that is curable if identified early enough. Despite this, many women acquire the cancer, which often has fatal consequences. Precancerous diseases that are detected early and treated promptly offer the best protection against cancer. The Papanicolaou (Pap) smear, the HPV DNA test, visual inspection of the cervix with acetic acid (VIA), and colposcopy are all techniques of cervical cancer screening. However, because the Pap smear is the most frequently used screening method worldwide and as it is the screening method employed by the Malaysian government [3], this study will focus on it.

In Malaysia, the Ministry of Health (MoH) and the National Population and Family Development Board (NPFDB) of the Ministry of Women and Family and Community Development provide cervical cancer public health measures including educational campaigns and advertisements, free Pap smear screening, and free HPV vaccination for 14-year-old schoolgirls.

According to the Malaysian Ministry of Health’s cervical cancer screening recommendation, all eligible women aged 20 to 65 who are sexually active should be screened for cervical cancer annually for the first three years. If she is considered to be at low risk for cervical cancer, she can now be tested once every three years. However, if she is considered to be at high risk, she should continue to get an annual pap smear. The number of new and current cases of cervical cancer has decreased dramatically during the previous 20 years, as have the fatalities from the disease. This is because the pap smear was introduced in the 1960s and all Malaysian native-born women can get free testing in public hospitals and health facilities [3].

However, these services do not apply to Yemeni women residing in Malaysia who, as a result of the recent instability and lack of security in Yemen, have chosen to relocate to Malaysia for educational and/or career possibilities, or to stay as immigrants. The health insurance is given for Yemenis in Malaysia if they are students or have working visa. There is no health insurance for immigrant Yemenis in Malaysia to cover free cancer-screening services, which adds to the problem. These women encounter various challenges in getting cervical cancer-related information and services that are available to Malaysian women since they are foreign immigrants. Barriers in language, culture, health beliefs, transportation, cervical cancer prices, and problems getting to and from healthcare facilities are all examples of these problems.

Even though cervical cancer is less common in most developing and developed countries because of screening that can detect the disease at an early stage, some groups, like immigrants, are still devastated by the disease. This is because the disease is less common because of screening that can detect the illness at an early stage [4].

Research carried out among Iraqi and African immigrant women in Malaysia showed that most of the responders exhibited a lack of awareness of cervical cancer and its screening with the Pap smear test. Many women had a hazy understanding of what a normal cervical smear entailed. One of the reasons Iraqi women in Malaysia didn’t get Pap smears was because they didn’t know about screening facilities as well as how much Pap smears cost [5]. African women in Malaysia who were married, had a regular health care providers (HCP), had knowledge, and perceived barriers were more likely to get a Pap smear [6].

Yemenis in Malaysia have reached a population of about 27,000 according to 2018 estimates of the Yemeni Embassy. Among these are Yemeni immigrant women who may not have any idea of cervical cancer screening services and lack the knowledge and good health attitudes in this regard. To date, there have been no studies regard to cervical cancer in Malaysia among Yemeni immigrant women.

As a result, it is necessary to investigate the prevalence of cervical cancer screening among Yemeni immigrants in Malaysia, as well as the factors that may influence their choice to get a Pap smear test. This will help more Yemeni immigrant women go to cervical cancer screenings. In addition, more people need to know about cervical cancer and how to get screened for it in order to cut down on the number of people who get it and the number of people who die from it.

The goal of this study is to fill in that gap by finding out how Yemeni immigrant women in the Klang Valley, Malaysia, are screened for cervical cancer and the factors affecting the screening status of Yemeni immigrant women in the Klang Valley, Malaysia.

## Materials and methods

### Study design and setting

A cross-sectional study was conducted on 355 Yemeni immigrant women in the Klang Valley, Malaysia. Participants must only be Yemeni immigrant women, aged 20 years old or older, who are married or have been married previously and who have a smart phone and the WhatsApp application. Those who had hysterectomy or were diagnosed with gynaecological cancer were not included in the study.

### Sampling technique and study subjects

A list of Yemeni immigrant women living in Klang Valley, Malaysia was obtained from the Yemeni embassy in Malaysia. A simple random sampling procedure using computer-generated process was used to pick participants from the list. The women in the list have been called and invited to participate in the survey. Interested women to participate in the study were validated for their eligibility based on the inclusion criteria.

### Study variables

#### Dependent variable

Cervical cancer screening practice

#### Independent variable

- Predisposing factors
  ■ Sociodemographic characteristics (age, level of education, marital status, employment and household monthly income),
  ■ Knowledge of cervical cancer and Pap smear, and
  ■ Perceived barriers
- Enabling factors
  ■ Insurance status,
  ■ Acculturation, and
  ■ Having a regular health care provider (family doctor), and
  ■ Access to health care facilities.

#### Operational definition

The operational definition for each variable is explained in Table 1.

**Table 1:** Operational definition of dependent and independent variables.

No.	Variable Terms	Definition	Measurement (coding used in data analysis)
**1**	Age	Respondent’s age counted in years.	1 = 20–34 2 = 35–49 3 = 50–65
**2**	Educational level	The highest education level completed by the respondents to date.	1 = Primary school 2 = Intermediate school 3 = Secondary school 4 = University or higher
**3**	Marital Status	Respondent’s status of being married, divorced or widowed.	1 = Married 2 = Divorced 3 = Widowed
**4**	Employment	Categorized as employed or unemployed.	1 = Unemployed 2 = Employed 3 = Retired
**5**	Household monthly income	The total income per month in Ringgit Malaysia from working family members who lives together in the house.	1 = ≤ RM 2000 2 = RM 2001–4000 3 = RM 4001 and above
**6**	Insurance status	Refers to whether respondents have health insurance or not for themselves.	1 = No 2 = Yes
**7**	Acculturation	Acculturation refers to is the process of culture change and adaptation that occurs when individuals with different cultures come into contact. For this study, It means speaking Malaysian language (Bahasa Melayu) or not.	1 = No 2 = Yes
**8**	Having a regular health care provider (family doctor)	Refer to regular visits to a health care providers (family doctor).	1 = No 2 = Yes
**9**	Knowledge	The level of knowledge of participants on cervical cancer and Pap smear test will be assessed by asking 6 questions. Questions are delivered to study participants in “Yes”, “No” or “I don’t know” options, and they are agreed or disagreed based on their awareness. Each correct response are given a score of 1 and a wrong answer or “I don’t know” is given a score of 0. The range of possible score is 0 to 6. Knowledge score <50% = poor knowledge, and >50% score = good knowledge [7].	1 = poor knowledge 2 = Good knowledge
**10**	Perceived barriers	Perceived barriers to screening will be assessed by using 5-point Likert-type scale ranging from strongly agree (5 points) to strongly disagree (1 point). The attitudinal statements were reported in the range of +1 to −1. The centre 0 corresponds to neutral response on Likert scale (neither agree nor disagree), +1 corresponds to totally agree and agree, and −1 corresponds to totally disagree and disagree. There were five attitude statements in the questionnaire so that the maximum score was +5 and the minimum score was −5. A negative score indicates having low perceived barrier, while positive and zero scores indicate having high perceived barriers regarding Pap smear screening [8].	1 = High barrier 2 = Low barrier
**11**	Practice	The practice will be assessed by asking respondents if they had attended a screening test for precancerous lesions at least once or if they had not attended in the past 3 years. Those who had gone for screening were considered to have practiced cervical cancer screening. Those who had not gone for screening were considered as not having practiced cervical cancer screening.	1 = No practice 2 = Practice
**12**	Immigrants	A person who has migrated from an origin or a place of birth to another destination across international borders with the intention of staying for a long period or permanently [9]. For this stud ]y, women who stay in Malaysia for a period of 5 months or more is considered as an immigrant.	

### Data collection method

The tool used to collect data was based on an online structured pre-tested questionnaire adapted from previous research [10]. The translation of the questionnaire into Arabic was done according to the Guidelines for Translation WHODAS 2.0 [11].

The final survey was created with Google Forms and distributed to the participants directly via WhatsApp. The questions are divided into four parts:

The first part of the questionnaire asks about a person’s age, level of education, marital status, employment, and household monthly income.

The second part examines participants’ knowledge of cervical cancer and screening. Six questions deal with knowledge predisposing factors. There are three options for each knowledge question: "yes," "no," or "I don’t know". Each right answer receives one point, while incorrect answers and those who reply "I don’t know" receive zero points. The potential scores range from 0 to 6. A score of one point was given if the correct answer was chosen and zero in the case of a wrong answer or “do not know” response. The knowledge was divided into good and poor based on 50% cut off.

The third section of the questionnaire is on the individuals’ practice. The questions are intended to assess the level of participation in the Pap smear screening by the participant. Respondents were asked if they had attended a screening test for precancerous lesions at least once in the previous three years or if they had not attended in the previous three years. Those who had attended the screening were regarded as practicing cervical cancer screening. Those who had not attended the screening were regarded as not practicing cervical cancer screening.

The fourth part of the questionnaire relates to the enabling factors (acculturation, insurance status, access to health care facilities, and having a health care provider (family doctor). The options were objective answers of "yes" and "no."

The final part contains questions about perceived barriers. Responses are given on a 5-point Likert-type scale, ranging from "strongly agree" (5) to "strongly disagree" (1) to statements about screening barriers. The attitudinal statements were reported in the range of +1 to −1. The centre 0 corresponds to neutral response on Likert scale (neither agree nor disagree), +1 corresponds to totally agree and agree, and −1 corresponds to totally disagree and disagree. There were five attitude statements in the questionnaire so that the maximum score was +5 and the minimum score was −5. A negative score indicates having low perceived barrier, while positive and zero scores indicate having high perceived barriers regarding Pap smear screening.

Face and content validity were checked and assessed by professional expert panellists from the Community Health Department at the Faculty of Medicine and Health Sciences, Universiti Putra Malaysia. The experts approved the educational material as efficient with respect to the study objectives. The translated questionnaire was pre-tested among 60 Yemeni women who were not participants in the study to get an understanding of the pitfalls that must be anticipated and dealt with. Cronbach’s alpha coefficient was used to assess the reliability of the rating scales’ internal consistency. Cronbach’s alphas for knowledge, enabling factors, and barriers that people thought about were 0.715, 0.717, and 0.79 respectively.

### Data analysis

The data obtained from the questionnaire was analysed using SPSS Version 25. All hypotheses tests are two-sided, with a level of significance of 0.05. The participating women’s sociodemographic characteristics, knowledge level, attitude toward, and practice of Pap smears were reported as frequencies, percentages, means, and standard deviations, as appropriate. Significant (P< 0.2) variables in bivariate analysis were entered into multivariate analysis and statistical significance was set at a p-value of <0.05 with a 95% confidence interval (CI). Factors that are included to get the best fitting model are (age, insurance status, knowledge, access to health care facilities and perceived barriers). Multicollinearity was checked to see the linear correlation among the independent variables by using standard error. Variables with a standard error of > 2 were dropped from the multivariate analysis. The result of the study was presented in tables, and texts.

### Ethical approval and consent to participant

Ethical clearance will be obtained from Jawatankuasa Etika Universiti Untuk Penyelidikan Melibatkan Manusia (JKEUPM) at Universiti Putra Malaysia [Ref No: UPM/TNCPI/RMC/JKEUPM/1.4.18.2 (JKEUPM)]. This study will follow the ethical criteria throughout its entire procedure. A participant consent form was taken by each participant prior to conducting the survey. Participants will be assured that all data will be used only for research purposes. Strict confidentiality is safeguarded throughout the study. Participants will be informed that they can withdraw from the study at any time before the completion of the study.

## Results

Data was obtained from 355 responses out of 600 invited to participate in the survey, yielding a response rate of 59 percent overall.

### Socio-demographic characteristics of respondents

The sociodemographic characteristics of participants are presented in Table 2. About half of responses, 178 (50.1%), were between the ages of 35 and 49, and just a few, 31 (8.7%), were between the ages of 50 and 65, while the rest, 146 (41.1%), were between 20 and 34 years old. The majority of them were married 306 (86.2%) and unemployed 251 (70.7%).

**Table 2:** Socio-demographics characteristics of the respondents (N = 355).

Variable	Frequency	Percentage (%)
**Age group**		
20–34 years	146	41.1
35–49 years	178	50.2
50–65 years	31	8.7
**Marital status**		
Married	306	86.2
Divorced	27	7.6
Widow	22	6.2
**Educational level**		
Primary	15	4.2
Intermediate	29	8.2
Secondary	114	32.1
University and above	197	55.5
**Employment status**		
Unemployed	251	70.7
Employed	97	27.3
Retired	7	2
**Household monthly income**		
≤ RM 2000	162	45.6
RM 2001–4000	118	33.2
RM 4001 and above	75	21.2

A total of 195 (54.9%) reported to have attained or undergone their university and above, about 138 (38.9%) have completed secondary education level. About 18 (5.1%) of people have at least intermediate education, and only 4 (1.1%) have primary school education.

Participants who have a household monthly income of ≤ RM 2000 were 162 (45.6%), and 118 (33.2%) have a household monthly income of RM 2001–4000 Malaysian Ringgit (RM).

### Knowledge of Pap smear and cervical cancer

Table 3 shows the distribution of respondents’ knowledge about Pap smears and cervical cancer. More than half of respondents 224 (63.1%) had heard of cervical cancer. Whereas, 70 (19.7%) believed that cervical cancer was caused by infection. A total of 113 (31.8%) respondents stated that cervical cancer is curable with early detection.

**Table 3:** Knowledge of Pap smear and cervical cancer (N = 355).

	Variables	Response		
		Yes n (%)	No n (%)	I do not know n (%)
1	Ever heard of cervical cancer	224 (63.1%)	122 (34.4%)	9 (2.5%)
2	Cervical cancer is associated with an infection	70 (19.7%)	218 (61.4%)	67 (18.9%)
3	Early detection of cervical cancer could make it curable	113 (31.8%)	104 (29.3%)	138 (38.9%)
4	Ever heard of a Pap smear	154 (43.4%)	190 (53.5%)	11 (3.1%)
5	Pap smear can detect changes in the cervix before they become cancer	135 (38.0%)	47 (13.2%)	173 (48.7%)
6	If someone has a normal Pap smear, she will not need more Pap smears in the future	149 (42.0%)	73 (20.6%)	133 (37.5%)

About 190 women (53.5%) had never heard of a Pap smear. Almost half of women (48.7%) did not know that a Pap smear can find changes in the cervix before they become cancerous, and 149 (42.0%) thought that if someone has a normal Pap smear, she will not need more Pap smears in the future. The respondent’s mean (SD) score for knowledge was 2.17 (1.33). Two hundred and eighty-six (80.6%) of the participants had low knowledge (score < 3), while 69 (19.4%) had good knowledge (score > 3).

### Practice of cervical cancer screening

Only 82 (23.1%) of respondents were practicing cervical cancer screening in the last the three years (Table 4).

**Table 4:** Practice of cervical cancer screening.

Variables	Category	Frequency	Percentage (%)
Have you ever screened for cervical cancer	Yes	112	31.5
	No	243	68.5
If yes when was the last time you screened	One year ago	23	6.5
	2–3 years ago	59	16.6
	More than 3 years ago	30	8.5

### Enabling characteristics of the respondents

The distribution of responses according to enabling variables is shown in Table 5. The majority of respondents, 261 (73.5%), stated that they do not have health insurance. The vast majority of responders 330 (93.0%) stated that they were unable to communicate in Bahasa Melayu (BM). Around 273 (76.9%) did not have a regular health care provider (HCP). 49 (13.8%) of respondents reported having no access to health care facilities in Malaysia where Pap smears are performed, whereas 306 (86.2%) reported having access to these health care facilities.

**Table 5:** Enabling factors (N = 355).

Variables	Frequency	Percentage (%)
**Insurance status**		
Yes	94	26.5
No	261	73.5
**Acculturation**		
Can speak Malaysian language (Bahasa Melayu (BM))	25	7.0
Cannot speak Malaysian language (Bahasa Melayu (BM))	330	93.0
**Having a regular health care provider**		
Yes	82	23.1
No	273	76.9
**Do you have an access to health care facilities?**		
Yes	306	86.2
No	49	13.8

### Perceived barriers to getting a Pap smear test

The distribution of respondents according to their perceived barriers to getting a Pap smear test is shown in Table 6. In all, more than 50% of respondents expressed uncertainty about the statement pertaining to their perceived barriers. The respondent’s mean (SD) score for perceived barrier to pap smear testing was 0.7 (1.6).

**Table 6:** Perceived barriers to getting Pap smear test (N = 355).

Item barriers	Strongly Agree n (%)	Agree n (%)	Neutral n (%)	Disagree n (%)	Strongly disagree n (%)
I am physically healthy, so I have no need for Pap smear	4 (1.1)	106 (29.9)	220 (62.0)	16 (4.5)	9 (2.5)
It is not important for a woman to have Pap smear	0 (0)	43 (12.1)	193 (54.4)	45 (12.7)	74 (20.8)
It is too embarrassing to do Pap smear	3 (0.8)	66 (18.6)	240 (67.6)	40 (11.3)	6 (1.7)
Pap smear is painful	3 (0.8)	88 (24.8)	248 (69.9)	9 (2.5)	7 (2.0)
Doing Pap smear will only make one worry	0 (0)	101 (28.5)	235 (66.2)	10 (2.8)	9 (2.5)
Lack of female screeners in health facilities is a reason for not doing Pap smear	2 (0.6)	84 (23.7)	247 (69.6)	13 (3.7)	9 (2.5)

About 284 (80%) of respondents had a high perceived barrier to getting a Pap screening test, whereas 71 (20%) had a low perceived barrier to getting a Pap smear test.

### Associations between predisposing factors, enabling factors, perceived barriers and the Pap smear test

Table 7 summarizes factors that are associated with and can predict cervical cancer screening uptake using a chi-square test of association and binary logistic regression. Significant (P< 0.2) variables in bivariate were entered into the final model for multivariate analysis and statistical significance was set at a p-value of <0.05 with a 95% confidence interval (CI).

**Table 7:** Factors associated with Pap smear uptake in the previous three years (N = 355).

Factors	Bivariate analysis					Multivariate analysis	
	Pap smear test within the previous three years n (%)		P-values	COR (95%CI)	P-values	AOR (95%CI)	P-values
	Yes	No					
**Age group**							
20–34	25 (17.1%)	121 (82.9%)	0.083		0.029	Ref	
35–49 years	48 (27.0%)	130 (73.0%)	0.036	1.79 (1.038–3.076)		1.47 (0.79–2.72)	0.225
50–65 years	9 (29.0%)	22 (71.0%)	0.131	1.98 (0.816–4.81)		5.39 (1.53–18.93)	0.009*
**Marital status**							
Married	76 (24.8%)	230 (75.2%)	0.165		0.354	Ref	
Divorced	3 (11.1%)	24 (88.9%)	0.121	0.38 (0.11–1.29)		0.66 (0.18–2.42)	0.528
Widow	3 (13.6%)	19 (86.4%)	0.25	0.48 (0.138–1.66)		0.33 (0.06–1.72)	0.189
**Educational level**							
Primary	2 (13.3%)	13 (86.7%)	0.64		0.217	Ref	
Intermediate	6 (20.7%)	23 (79.3%)	0.55	1.69 (0.3–9.65)		2.78 (0.39–20)	0.309
Secondary	24 (21.1%)	90 (78.9%)	0.488	1.73 (0.37–8.21)		5.12 (0.82–32.03)	0.081
University	50 (25.4%)	147 (74.6%)	0.307	2.21(0.48–10.14)		5.5 (0.93–32.62)	0.061
**Employment status**							
Unemployed	59 (23.5%)	192 (76.5%)	0.85		0.365	Ref	
Employed	22 (22.7%)	75 (77.3%)	0.87	0.95 (0.55–1.67)		0.9 (0.46–1.74)	0.746
Retired	1 (14.3%)	6 (85.7%)	0.54	0.54 (0.064–4.59)		0.14 (0.01–2.15)	0.160
**Household monthly income**							
≤ RM 2000	34 (21.0%)	128 (79.0%)	0.62		0.746	Ref	
RM 2001–4000	28 (23.7%)	90 (76.3%)	0.59	1.17 (0.66–1.07)		0.94 (0.49–1.82)	0.863
RM 4001 and above	20 (26.7%)	55 (73.3%)	0.33	1.37 (0.73–2.59)		0.74 (0.34–1.62)	0.451
**Knowledge**							
Low	45 (15.7%)	241(84.3%)	<0.001	6.2 (3.50–10.95)	0.000	Ref	
High	37 (53.6%)	32 (46.4%)				6.67 (3.45–12.9)	0.000*
**Insurance status**							
No	54 (20.7%)	207 (79.3%)	0.74	1.63 (0.953–2.77)	0.018	Ref	
Yes	28 (29.8%)	66 (70.2%)				2.22 (1.15–4.3)	0.018*
**Acculturation**							
Cannot speak Malaysian language (Bahasa Melayu (BM))	75 (22.7%)	255 (77.3%)	0.55	1.32 (0.53–3.3)	0.921	Ref	
Can speak Malaysian language ((Bahasa Melayu (BM))	7 (28.0%)	18 (72.0%)				0.94 (0.3–2.98)	0.921
**Having health care provider (HCP)**							
No	59 (21.6%)	214 (78.4%)	0.23	1.41 (0.81–2.5)	0.940	Ref	
Yes	23 (28.0%)	59 (72.0%)				0.97 (0.49–1.93)	0.940
**Access to health care facilities**							
No	3 (6.%)	46 (93.9%)	0.01	5.34 (1.65–17.64)	0.019	Ref	
Yes	79 (25.8%)	227 (74.2%)				4.64 (1.29–16.65)	0.019*
**Perceived barrier**							
Low	28 (39.4%)	43 (60.6%)	<0.001	2.8 (1.6–4.9)	0.006	Ref	
High	54 (19.0%)	230 (81.0%)				2.5 (1.3–4.83)	0.006*

* Statistically significant

According to findings from regression analysis, the respondents aged 50–65 years (AOR = 5.39, 95% CI: 1.53–18.93) are more likely to have a good Pap smear uptake than younger respondents (aged 20–34). Insurance status was a significantly associated factor for having good Pap smear uptake. The odds of having good Pap smear uptake among the respondents who have insurance were 2.2 times (AOR = 2.22, 95% CI = 1.15–4.3) higher than respondents who do not have insurance. Knowledge was also significantly associated with Pap smear uptake within three years. When compared to women with poor knowledge, those with high knowledge are more likely to have a good Pap smear uptake (AOR = 6.67, 95% CI = 3.45–12.9). Access to health care facilities was one of the significant factors of Pap smear uptake. Women who have access to health care facilities are more likely to get Pap smears (AOR = 4.64, 95% CI = 1.29–16.65) than those who do not have access to health care facilities. The odds of having Pap smear uptake in the previous three years among those having high perceived barrier was (AOR = 2.5, 95% CI = 1.3–4.83) higher than those having low perceived barrier.

## Discussion

The finding of the study suggests that cervical cancer screening uptake was 23.1% overall among Yemeni immigrants in Malaysia during the previous three years. This finding is lower than the studies done among African (24%) and Iraqi (27.2%) female immigrants in Malaysia [5, 6]. It is also lower than cervical cancer practice in Malaysia, which according to a 2006 national survey conducted by the institute for public health (IPH), 43.7% of women in Malaysia have undergone the pap smear test, which is higher than the 1996 results of 26% and 12.8% in 2011, respectively. The most recent report in 2019 indicated that 36.6% of women had undergone the test, although it was still below the recommended coverage of 80% [12]. This might be due to low level of knowledge about cervical cancer and its screening as only (40.3%) of the respondents were knowledgeable on cervical cancer and Pap smear or due to limited access of screening services. The low level of knowledge and prevalence rate of cervical cancer screening practice may justify the need to have a specific intervention program to reach the targeted group and improve their knowledge.

The study showed that (63.1%) they have never heard of cervical cancer and only (19.7%) know that it can be caused by infection. In addition (53.5%) had never heard of a Pap smear. This is consistent with the study done in Malaysia among African immigrant women in which more than half of the respondents (60.3%) reported that they have never heard of cervical cancer, only 31 (9.7%) knows that HPV causes cervical cancer and (67.2%) never heard of Pap smear [6].

Several studies show that immigrants are more vulnerable to cervical cancer. Some of them done in high-income countries indicated that migrant women had a higher incidence of cervical cancer than local equivalents. A cross-sectional study using merged data from four nationwide registries in Norway concluded Immigrants had lower rates of participation compared with Norwegian-born women; Western Europe [adjusted odds ratio (OR), 95% confidence interval (CI): 0.84, 0.81–0.88], Eastern Europe (OR 0.64, 95% CI: 0.60–0.67), Asia (OR 0.74, 95% CI: 0.71–0.77), Africa (OR 0.61, 95% CI: 0.56–0.67) and South America (OR 0.87, 95% CI: 0.79–0.96) [13]. Furthermore, migrant women have lower cervical cancer screening participation rates, which raises the likelihood of being detected at later stages of the disease and has a detrimental influence on treatment results [14]. There was a large variation in the proportion of non-adherent immigrants by country of origin and region. Being unemployed, unmarried, with a low income, and having a male general practitioner were all connected to non-adherence, regardless of where they came from [15].

Age, marital status, employment, household monthly income, acculturation, insurance status, regular health care provider, access to health facilities, knowledge, and barriers were all investigated as potential predictors of cervical cancer screening uptake. According to this study, age group 50–65 (p = 0.009), insurance status (p = 0.018) and knowledge (P<0.001) are significantly associated with cervical cancer screening uptake. This finding is consistent with a cross-section study that was done among immigrant and refugee women in Ohio and revealed that there was a statistically significant association between screening age (p < 0.001), insurance status (p = 0.033), and general knowledge of cervical cancer (p = 0.038) with cervical cancer screening [16].

Older respondents in this study, aged (50–65 years), are more likely than younger respondents to have a significant Pap smear uptake. Another register-based cohort research in Finland found a substantial reduction in cervical cancer mortality among women asked for screening in Helsinki at the age of 65 [17].

Good cervical cancer screening practices in this study were found among those having high perceived barriers (AOR = 2.5, 95% CI = 1.3–4.83) than those having low perceived barrier. A systematic review was conducted among immigrant women in Australia and illustrated the factors influencing women’s desire to take part in screening, including low-risk perception, insufficient knowledge, and unavailability of female health professionals, as key barriers [18].

The current study showed that cervical cancer screening uptake is also significantly influenced by access to health care facilities. Those who have access to health care facilities are five times more likely to have an uptake than those who do not have access to health care facilities. Accessibility to health care may be determined in both a spatial and aspatial context. The geographical constraint that limits one’s ability to access care is referred to as “spatial access”. Distance to the screening site was associated with a higher rate of non-adherence [19].

Non-geographical barriers limiting access to care, often evaluated by socioeconomic variables, are referred to as aspatial access. A primary healthcare register-based study in Norway found that higher income, younger age, and residence in rural areas were associated with Pap smear uptake among immigrants [13].

A study was conducted among African immigrant women residing in Brisbane, Australia, and indicated the importance of cultural factors in promoting cervical cancer screening and concluded that providing culturally suitable approaches to cervical screening practices among health workers who use service delivery approaches that are sensitive to group cultures was identified as a facilitator of cervical screening [20]. In addition, direct and indirect economic costs, such as lost income, transportation costs, and waiting time, are all perceived challenges for screening attendance [21, 22].

As mentioned earlier our study was compared more with studies done among immigrant women in other countries because we could not find much published studies done among immigrant women in Malaysia and to the knowledge of the researcher, this is the first study conducted among Yemeni immigrant women in Malaysia that identified the prevalence of cervical cancer screening practices and the factors influencing them. The current study was conducted using a cross-sectional approach, and no causal relationship could be demonstrated. Apart from that, because this study relies on self-reported Pap smear practice rather than clinician or laboratory records, recall bias may exist. Finally, and perhaps most importantly, this study has the problem of not being generalizable to other Yemenis in other places.

In conclusion, the participants’ overall prevalence of cervical cancer screening was low. Age group 50–65, insurance status, knowledge of cervical cancer and Pap smears, access to health care facilities and perceived barriers were the predictors of Pap smear uptake that were determined in this study. Efforts to increase immigrant women’s participation in cervical cancer screening must target barriers to access to healthcare services. In addition, increasing knowledge about cancer of the cervix and its screening is important for screening uptake in order to reduce the incidence and mortality associated with cervical cancer. As a result, intervention studies and programs are needed to increase awareness and practice of cervical cancer screening services in this study group.

## Supporting information

S1 File English version questionnaire. (DOCX)

S1 Dataset SPSS data set. (SAV)
