# Supplementary material for: Factors affecting cervical cancer screening among Yemeni immigrant women in Klang Valley, Malaysia: A cross sectional study
Source: PLoS One. 2023 Dec 15;18(12):e0290152. doi: 10.1371/journal.pone.0290152 (PMC10723656; doi:10.1371/journal.pone.0290152)
Supplement: S1 File — (DOCX) [file pone.0290152.s001.docx]

**Study Questionnaire**

Please answer the following questions by filling in the blanks or tick (√) the appropriate answer

**Part I: Sociodemographic factors:**

|  | Question |  |
| --- | --- | --- |
| 1 | What is your age? |  |
| 2 | What is your current marital status? | 🞎 Married  🞎 Divorced  🞎 Widow |
| 3 | What is your level of education? | 🞎 Primary school  🞎 Intermediate school  🞎 Secondary school  🞎 University or higher |
| 4 | Are you currently: | 🞎 Unemployed  🞎 Employed  🞎 Retired |
| 5 | How much is your household monthly income (MYR)? |  |

**Part II: Knowledge on cervical cancer and Pap smear test**

This section consists of questions which include knowledge on cervical cancer and knowledge on Pap smear test. Tick (√) the appropriate answer. If you are unsure about how to answer a question, please give the best answer you can.

|  | Variables | Yes | No | I do not know |
| --- | --- | --- | --- | --- |
| 1 | Ever heard of cervical cancer |  |  |  |
| 2 | Cervical cancer is associated with an infection |  |  |  |
| 3 | Early detection of cervical cancer could make it curable |  |  |  |
| 4 | Ever heard of a Pap smear |  |  |  |
| 5 | Pap smear can detect changes in the cervix before they become cancer |  |  |  |
| 6 | If someone has a normal Pap smear, she will not need more Pap smears in the future |  |  |  |

**Part III: Prevalence of Pap smear uptake in the past three years**

This part consists of a question on the time of the last Pap smear uptake that has been done in the past three years. Tick (√) the appropriate answer

| Question | Response |
| --- | --- |
| Have you had cervical cancer screening before? | 🞎Yes  🞎No |
| If yes, when was your last Pap smear test done? | 🞎 Never had Pap smear test before  🞎 One year ago  🞎 2-3 years ago  🞎 More than 3 years ago |
|  |  |
|  |  |
|  |  |

**Part IV: Enabling Factors**

This section consists of questions which include some enabling factors for Pap smear test practice. Tick (√) the appropriate answer. If you are unsure about how to answer a question, please give the best answer you can.

|  | Question | Yes | No | I don’t know |
| --- | --- | --- | --- | --- |
| 1 | Have a medical insurance |  |  |  |
| 2 | Speak Malaysian language (Bahasa Melayu) |  |  |  |
| 3 | Have a regular health care provider (family doctor) |  |  |  |
| 4 | Have an access to health care facilities |  | | |

**Part V: Perceived Barriers to Having Pap Smear Test**

The following sentences are some ideas related to the Pap smear test. Please tick (√) next to the answer that best describes your belief. There is no right or wrong answer. Therefore, if you are unsure or do not know an answer, feel free to answer what you believe.

|  | Variables | **Strongly Disagree** | **Disagree** | **Neutral** | **Agree** | **Strongly Agree** |
| --- | --- | --- | --- | --- | --- | --- |
| 1 | I am physically healthy, so I have no need for Pap smear |  |  |  |  |  |
| 2 | It is not important for a woman to have Pap smear |  |  |  |  |  |
| 3 | It is too embarrassing to do Pap smear |  |  |  |  |  |
| 4 | Pap smear is painful |  |  |  |  |  |
| 5 | Doing Pap smear will only make one worry |  |  |  |  |  |
| 6 | Lack of female screeners in health facilities is a reason for not doing Pap smear |  |  |  |  |  |
